# Mitochondrial phylogeny of the brittle star genus *Ophioderma*

**DOI:** 10.1038/s41598-022-08944-0

**Published:** 2022-03-29

**Authors:** H. A. Lessios, Gordon Hendler

**Affiliations:** 1 Smithsonian Tropical Research Institute, Box 0843-03092, Balboa, Panama; 2 Natural History Museum of Los Angeles County, 900 Exposition Boulevard, Los Angeles, CA 90007 USA

**Keywords:** Evolution, Evolutionary genetics, Molecular evolution, Speciation

## Abstract

We reconstructed the mitochondrial phylogeny of the species of the brittle star genus *Ophioderma*, using sequences of the Cytochrome Oxidase I gene (COI) to address four questions: (i) Are the species of *Ophioderma* described on morphological evidence reflected in mitochondrial genealogy? (ii) Which species separated from which? (iii) When did speciation events occur? (iv) What is the rate of COI evolution in ophiuroids? We found that most of the 22 described species we sampled coincide with monophyletic clusters of COI sequences, but there are exceptions, most notably in the eastern Pacific, in which three undescribed species were indicated. The COI phylogeny lacks resolution in the deeper nodes, but it does show that there are four species pairs, the members of which are found on either side of the central American Isthmus. Two pairs with a genetic distance of ~ 4% between Atlantic and Pacific members were probably split during the final stages of Isthmus completion roughly 3 million years ago. The rate of divergence provided by these pairs allowed the calibration of a relaxed molecular clock. Estimated dates of divergence indicate that the lineages leading to extant species coalesce at times much older than congeneric species in other classes of echinoderms, suggesting that low extinction rates may be one of the reasons that ophiuroids are species-rich. The mean rate of COI substitution in *Ophioderma* is three times slower than that of echinoids. Conclusions of previous mitochondrial DNA studies of ophiuroids that relied on echinoid calibrations to determine divergence times need to be revised.

## Introduction

The Ophiuroidea (brittle stars), with more than 2000 species, is one of the two most species-rich echinoderm classes^1^. They inhabit benthic environments in nearly all depths and latitudes^2^. Molecular data have been used to elucidate their higher level phylogeny^3–9^, to delimit species borders^10–19^, and to document intraspecific population genetic structure^20–26^. However, to our knowledge, there are only two published molecular phylogenies of brittle stars at the genus or family level, those of *Macrophiothrix*^27^ and of ophiocomid brittle stars^28^. Phylogenies showing the order of splitting of congeneric species from each other, the time that these splits occurred, and sister species relationships are the first step towards determining the possible causes of speciation. This paper attempts to make the first strides towards this end through a mitochondrial phylogeny of the species of the genus *Ophioderma*.

The genus *Ophioderma* Müller & Troschel, 1840 encompasses 33 extant described species^1^, though two, *O. tonganum* Lütken, 1872 and *O. propinquum* Koehler, 1895, both described from the Indo-Pacific, appear to be based on doubtful locality information and possibly misidentified^29–31^. Another species, *O. besnardi* Tommasi, 1970, described from Brazil^32^ may be the juvenile form of *O. cinereum*^33^. Five of the 33 species were recently described. Stöhr et al.^34^ split *O. longicaudum* (Bruzelius, 1805) into *O. longicaudum, O. zibrowii* Stöhr, Weber, Boissin & Chenuil, 2020*, O. hybridum* Stöhr, Weber, Boissin & Chenuil, 2020, and *O. africanum* Stöhr, Weber, Boissin & Chenuil, 2020 and also resurrected *O. guineense* Greeff, 1882, which Madsen^35^ had placed into synonymy with *O. longicaudum*. Granja-Fernandez et al.^36^ described *Ophioderma hendleri* Granja-Fernandez, Pineda-Enriquez, Solis-Marin & Laguarda-Figueras, 2020 and pointed out that a number of *Ophioderma* museum specimens from the eastern Pacific were erroneously identified as belonging to previously described species.

The species of *Ophioderma* are distributed on both sides of tropical and subtropical America, the Mediterranean, and the West African coast. *O. anitae* Hotchkiss, 1982*, O. appressum* (Say, 1825)*, O. brevicaudum* Lütken, 1856*, O. brevispinum* (Say, 1825)*, O. cinereum* Müller & Troschel, 1842*, O. devaneyi,* Hendler & Miller, 1984,* O. elaps* Lütken, 1856*, O. ensiferum* Hendler & Miller, 1984*, O. guttatum* Lütken, 1859*, O. holmesii* (Lyman, 1860)*, O. pallidum* (Verrill, 1899)*, O. phoenium* H.L. Clark, 1918*, O. rubicundum* Lütken, 1856*,* and *O. squamosissimum* Lütken, 1856 are found in the Caribbean, Bahamas, and Bermuda^37,38^. Of these, only *O. brevispinum*, *O. cinereum* and (perhaps) *O. brevicaudum* have a range extending south to Brazil^39,40^, and only *O. brevicaudum* and *O. brevispinum* spread north to the Carolina Banks and to the Cape Cod respectively^38^. *O. januarii* Lütken, 1856 is common in Brazil^40–43^. A nominal Brazilian species, *O. divae* Tommasi, 1971was described from Baía de Santos in Sao Paulo State^44^, but it has never been reported from any other location. *O. longicaudum* exists in the Eastern Atlantic from Bretagne to Macaronesia and widely in the Mediterranean^34,45^. *O. zibrowii* is found in the eastern Mediterranean. *O. hybridum* is only known from the coast of Tunisia. *O. africanum* is only known from Senegal. *O. guineense* is listed by Stöhr et al.^34^ as extending from Senegal to the Gulf of Guinea, although one mitochondrial clade, designated as corresponding to this species, was found by Boissin et al.^14^ to also be present in the Mediterranean. *O. wahlbergii* Müller & Troschel, 1842 is found on the Atlantic coast of South Africa from Namibia to Danger point^46,47^. Depth records of most Atlantic species range from the littoral to ca 200 m, but *O. pallidum* has only been reported from 198 to 360 m off Cuba^38^.

In the eastern Pacific there are 8 nominal species: *Ophioderma panamense* Lütken 1859*, O. pentacanthum* Clark 1917*, O. teres* (Lyman 1860)*,* and *O. variegatum* Lütken 1856 range from southern California to Ecuador at depths of 0 to approximately 100 m^48^ *O. hendleri* is spread from the Gulf of California to Colombia^36^. Other species have more restricted reported ranges. *O. vansyoci* Hendler 1996 is known from only two localities, one on each side of Baja California^49,50^*, O. peruanum* Pineda-Enriquez, Solis-Marin, Hooker & Laguarda-Figueras, 2013 is only known from the coast of Peru^30^*,* and *O. sodipallaresi* Caso, 1986 is only known from Mazatlán, Mexico^51^. *O. elaps*, known from the Caribbean^29,31,52–54^, where it was originally collected and described, has also been reported from one locality in the Galapagos^29,48,55^.

Fossils ascribed to extinct species of *Ophioderma* have been reported from the Permian^56^, Triassic^56–59^, Jurassic^60–62^, Cretaceous^63^, and the Miocene^64^. Chen and McNamara^57^ have pointed out that many of these records are based on disarticulated plates or possess characters that do not place them in the genus. Aronson^65^, on the other hand, described Jurassic fossils of *Ophioderma* at the British museum as “exceptionally well preserved”. The transcriptomic phylogeny of O'Hara, et al.^5^, calibrated by multiple reliably identified fossils, suggests that the Ophiodermatidae did not originate until the Cretaceous, approximately 100 MYA (million years ago).

The majority of species of *Ophioderma* have lecithotrophic vitellaria larvae that in the laboratory settle approximately in eight days^37,66–70^. However, *O. wahlbergii* off South Africa broods its young^71,72^, as do individuals previously considered as belonging to *O. longicaudum* but recently described as *O. zibrowii* from the eastern Mediterranean and *O. hybridum* from Tunisia^14,15,34^. Individuals of some species are estimated to be quite long-lived; *O. brevispinum* is thought to reach an age of 25–28 years, and *O. longicaudum* 30 years^69^.

In this study we use partial sequences of the Cytochrome Oxidase I (COI) mitochondrial gene to address the following questions: (i) Are the species of *Ophioderma* described on morphological evidence reflected in mitochondrial genealogy? (ii) Which species separated from which? (iii) When did speciation events occur in this genus? (iv) What is the rate of COI evolution in ophiuroids?

## Results and discussion

**Figure 1:**
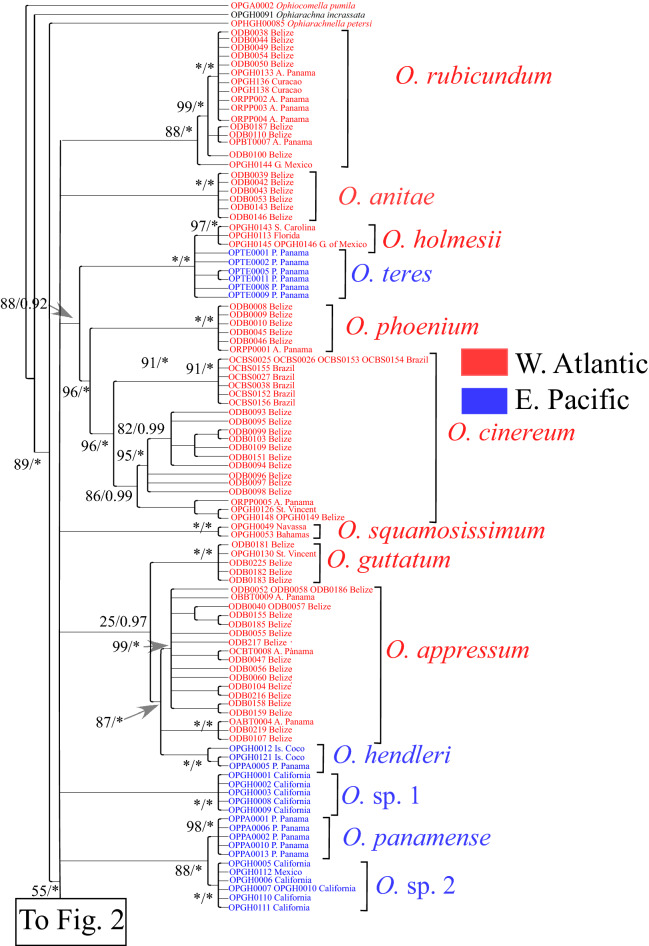
Part of the phylogeny of COI haplotypes of *Ophioderma*. Second part is shown in Fig. 2. Reconstruction is based on Maximum Likelihood (ML), using RAxML^109^, and Bayesian Information (BI) using MrBayes^108^. The tree is rooted on *Ophiocomella pumila*, but *Ophiarachna incrassata* and *Ophiarachnella petersi* were also included in the phylogeny to verify monophyly of *Ophioderma*. All nodes that did not receive support of at least 80% in ML or 0.9 in BI have been collapsed. Numbers next to nodes indicate ML and BI support (in this order). Asterisks indicate support of 100%. Codes indicate individuals in which a haplotype was present.

**Figure 2:**
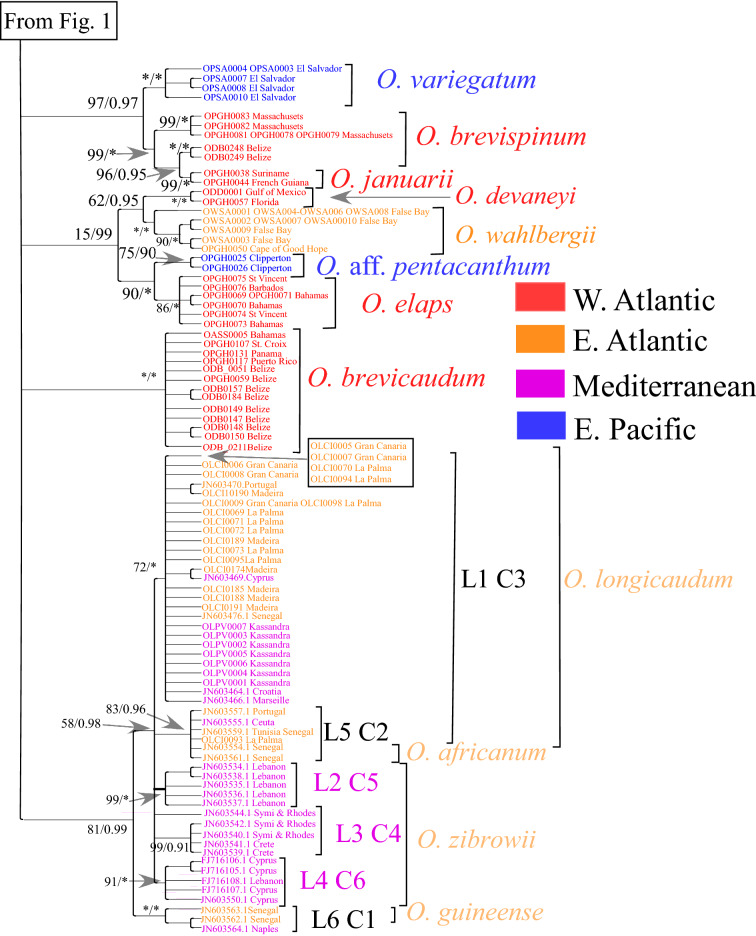
Second part of the phylogeny of COI haplotypes of *Ophioderma*, continued from Fig. 1. All nodes that did not receive support of at least 80% in ML or 0.9 in BI have been collapsed. Numbers next to nodes indicate ML and BI support (in this order). Codes indicate individuals in which a haplotype was present. Asterisks indicate support of 100%. Haplotypes obtained from the study of Boissin, et al.^14^ are identified by codes that start with either “JN” or “FJ” (GenBank numbers). Clade labels starting with “L” are those designated by Boissin, et al.^14^ , labels starting with C are from Weber et al.^101^.

The COI phylogeny of *Ophioderma* (Figs. 1 and 2) showed little resolution in deeper nodes. Thus, little can be said about the relationships between species on COI alone. However, COI of most morphologically recognized species was shown to be monophyletic, which bolsters the case that COI evidence can be used to delimit species in this genus. Part of the doubt created by the Maximum Likelihood (ML) reconstruction is that the genus *Ophioderma* may not be monophyletic with respect to *Ophiarachnella*. The node joining these two genera was supported with a posterior of 1.00 in MrBayes, but with only 55% of the bootstrap reiterations of RAxML (Fig. 1). Conversely, the node joining these two genera with *Ophiarachna* received support of 1.00 in MrBayes and of 89% in RAxML. Such discrepancies between methods of phylogenetic reconstruction have made it necessary to collapse nodes that did not receive high support from at least one of the two methods.

### Species delimitation

COI sequences of most morphologically defined species were monophyletic, but there were some inconsistences between morphology and DNA. In the Atlantic, two COI haplotypes, one from French Guiana and one from Suriname, which were morphologically identified as belonging to *O. januarii,* were nested within haplotypes of *O. brevispinum*. That this was not the result of low phylogenetic resolution is evident from their being reciprocally monophyletic from haplotypes of *O. brevispinum* from Belize, while the Belize and South American clades were sister to *O. brevispinum* from Massachusetts (Fig. 2). *O. januarii* and *O. brevispinum* are morphologically similar^74^, but the arms of the former are broader and more carinate near the disk and more tapered distantly, and the arm spines are longer and more tapered^73,75^. It remains to be determined whether they are, indeed, separate species. Another possibility is that Caribbean *O. brevispinum* and South American *O. januarii* belong to the same species, whereas the Massachusetts “*O. brevispinum”* is a separate species. This hypothesis would be consistent with a strict interpretation of relative genetic distances. The Maximum Composite Likelihood Genetic Distance between Caribbean *O. brevispinum* and *O. januarii* (6.80%) is smaller than the genetic distance between Massachusetts and Caribbean *O. brevispinum* (9.53%) or between Massachusetts *O. brevispinum* and *O. januarii* (8.05%). It is also possible that all three clades are separate species. Although molecular sampling in Brazil is necessary to choose between these alternatives, it would not be surprising if North American populations ascribed to *O. brevispinum*, occurring at higher latitudes than any other species of *Ophioderma*, were a distinct species. These northern populations had, in fact, been described as belonging to *O. olivaceum* by Ayres in 1852^76^, but Lyman^77^, without providing an explanation, synonymized *O. olivaceum* with “*Ophiura brevispina”.*

In the eastern Pacific there were two distinct and distantly related COI clades, the morphology of which does not fit the species description of any species from this ocean. One clade, designated in Fig. 1 as *O*.sp.1 and found in Anacapa, San Clemente and Santa Barbara islands off California, has a genetic distance of 14.31% from a clade found in California and Mexico that is designated as *O.* sp. 2, which it resembles in gross morphology (Supplementary Table S1). The latter clade, is sister to *O. panamense*, but separated by a genetic distance of 10.53% from it. Given the magnitude of these genetic distances, these two clades in all probability represent undescribed species. The morphology of the specimens from which the sequences were obtained clearly indicates that they do not belong to *O. pentacanthum, O. vansyoci, O. peruanum,* or *O. sodipallaresi*, nominal eastern Pacific species which we were unable to sequence. Two haplotypes of *Ophioderma* from Clipperton are reciprocally monophyletic with those of Atlantic *O. elaps* (Fig. 2). Given that their genetic distance from *O. elaps* is 4.95%, slightly higher than the distance between *O. holmesii* and *O. teres* (Supplementary Table S1), and given that *O. elaps* has also been reported from the eastern Pacific^29^, they might have been expected to belong to this species. However, they are morphologically very different from *O. elaps* and much more similar to *O. pentacanthum.* They differ from *O. pentacanthum* in that their arm spines are longer and more tapered, their dorsal arm plates are trapeziform and relatively narrow, their ventral arm plates are W-shaped, rather than rhomboid or quadrangular, and separated by large gaps. These specimens must also belong to an undescribed species to which we refer here as *O.* aff. *pentacanthum.*

### Relationships between species

Despite the low resolution of the COI phylogenetic reconstruction, evidence of shared common ancestors among some species is recorded in this mitochondrial marker (Figs. 1 and 2). The common ancestor of *O. holmesii* and *O. teres* split from the common ancestor of *O. phoenium* and *O. cinereum*. That *O. phoenium* and *O. cinereum* are sister to each other was also shown by Bribiesca-Contreras et al.^8^, based on sequence from1462 exons and from COI, and by Christodoulou et al.^9^ based on the same data, plus sequence from 28S. These studies did not sample *O. holmesii* but reported that *O. peruanum*, which we did not sample, is sister to *O. teres*. It is, therefore, possible that the eastern Pacific *O. teres* and *O. peruanum* are closely related and their ancestor was separated from the Atlantic *O. holmesi* by the rise of the Isthmus of Panama. Contrary to the unsupported speculation of Madsen^35^, and in accordance with the conclusions of Tortonese^45^, which were based on several reliable morphological features, *O. cinereum* and the *O. longicaudum* complex, with an average genetic distance of 12.43% from each other (Supplementary Table S1) are not related. *O. guttatum* is an outgroup of *O. appressum* and *O. hendleri*. In the phylogeny of Bribiesca-Contreras et al.^8^ (which does not include *O. hendleri*)*, O. guttatum* is an outgroup to a clade composed of *O. squamosissimum* and *O. vansyoci*, but the lower resolution of COI in our data did not provide support for the node joining it with *O. squamosissimum*. *O. appressum* and *O. hendleri* are another amphi-isthmian pair, but probably separated earlier than the completion of the Isthmus (see below). The members of the majority of geminate species in a variety of organisms were either separated by the protracted emergence of the central American Isthmus before the final closure, or else appear as if they have done so because of the extinction of true geminates^78,79^. Bribiesca-Contreras et al.^8^ show *O. variegatum*, rather than *O. hendleri,* as the sister species of *O. appressum*, but, as *O. hendleri* had not yet been described at the time of their publication^36^, it is not unexpected that they would assign the specimen they sequenced to *O. variegatum.* As Granja-Fernandez et al.^36^ point out, *O. hendleri* and *O. variegatum* can easily be confused, because they both have radial shields covered with granules and naked adoral shields. The Isthmus also appears to have separated *O. variegatum* (proper) from the common ancestor of *O. brevispinum* and *O. januarii*, and also *O.* aff. *pentacanthum* at Clipperton from Atlantic *O. elaps* (Fig. 2). *O. wahlbergii* in both the Bribiesca-Contreras et al.^8^ and the Christodoulou et al.^9^ phylogenies appears as an outgroup of nearly all other species of *Ophioderma,* which is not incompatible with our COI phylogeny that lacks support for deep nodes. The COI phylogeny, however, shows *O. wahlbergii* to be in a clade that includes *O. devaneyi* and *O. elaps* and *O.* aff. *pentacanthum,* species that were not included in the Bribiesca-Contreras^8^ et al. and the Christodoulou et al.^9^ phylogenies.

We have included in our set of data sequences of *O. longicaudum* from a study by Boissin, et al.^14^ primarily to determine the affinities of our sample from the Kassandra peninsula in the northern Aegean to the six clades they discovered. Stöhr, et al.^15^, Boissin, et al.^14^ and Weber et al.^16,80^ found that individuals with brooded young belonged to mitochondrial clades labeled L2, L3 and L4 (as defined in ref.^14^), all found in warm, oligotrophic waters of the Mediterranean east of Peloponnese, whereas lineages L1, L5, and L6 contained broadcast spawning individuals. They suggested that the brooding lineages are separate species, and they were subsequently described as such^34^. In our phylogeny (Fig. 2) the Kassandra specimens were nested in lineage L1 along with specimens from the eastern Atlantic, Cyprus, Croatia, and Marseille. Their average genetic distance from the rest of clade L1 is 0.62%, whereas from the other clades of the *O. longicaudum* complex their average distance is 4.39% (Supplementary Table S1). Clade L1is predominantly found in the western Mediterranean but also in the Saronic Gulf, east of the Peloponnese^14^. Its presence at the Kassandra peninsula in the northern Aegean may be related to the colder waters of this area, because this clade is adapted to lower temperatures^80^.

### Chronology of branching and rate of evolution

**Figure 3:**
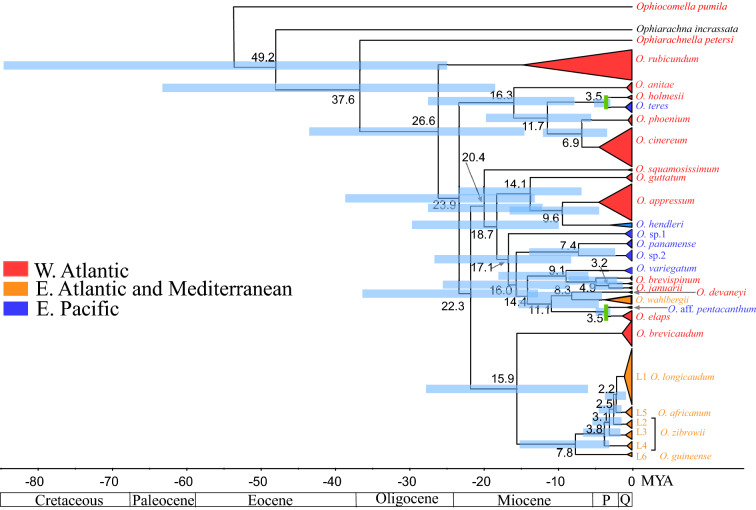
Chronogram of *Ophioderma* generated by BEAST v. 1.10.4^111^, showing median and 95% Highest Posterior Density (HPD) times of divergence between the clades. A log-normal uncorrelated relaxed clock was calibrated with an exponential prior that assumes that the minimum time of splitting between *O. holmesii* and *O. teres* and also between *O. elaps* and *O*. aff*. pentacanthum* (marked with green bars) was the completion of the Central American Isthmus 3 million years ago. Numbers next to nodes indicate median estimates of divergence times.

In the COI phylogeny (Figs. 1 and 2), there were four clades separated by the Central American Isthmus: (a) Sequences of *O. appressum* were nested in those of *O. hendleri* (Fig. 1); (b) *O. variegatum* was reciprocally monophyletic with the *O. brevispinum-O. januarii* clade (Fig. 2); (c) Sequences of *O. holmesii* were nested in those of O*. teres* (Fig. 1)*;* and (d) Sequences of *O. elaps* were sister to sequences of *O*. aff*. pentacanthum* (Fig. 2). The genetic distance between members of pair (a) was 13.03%, the distance between members of pair (b) was 12.99%, whereas that between members of pair (c) was 4.02% and between members of pair (d) was 4.95% (Supplementary Table S1). We, therefore, assumed that the divergence between the members of the last two pairs was more likely to reflect separation caused by the final stages of the emergence of the Central American Isthmus roughly 3 million years (MY)^79^. Thus, the split of *O. holmesii* from *O. teres* and the split of *O. elaps* from *O.* aff. *pentacanthum* were given an offset of 3 MY, and the resultant rate of COI divergence in *Ophioderma* was used by BEAST to estimate dates of clade separation by a log normal relaxed clock (Fig. 3). To estimate divergence times, it is necessary to preserve information on branch length. Nodes, if collapsed because of low support, would produce a false impression of antiquity. For this reason, all nodes in the fully resolved RAxML tree needed to be included in the BEAST analysis, even when they might have received low support. Figure 3, therefore, constitutes a chronogram. Given the wide 95% Highest Posterior Density (HPD) intervals of each node, the chronogram is compatible with the collapsed phylogenetic tree (Figs. 1 and 2).

The general impression provided by the BEAST chronology (Fig. 3) is that in comparison to echinoid and asteroid molecular phylogenies, clades leading to extant species of *Ophioderma* are quite old. In fact, they may be much older than is estimated by median ages in COI, because codon positions of mitochondrial DNA (mtDNA) that are free to vary are likely to become saturated with time. That COI sequences underestimate the antiquity of the older clades is revealed by a comparison of the age of deeper nodes of *Ophioderma* involving the outgroups, with the estimated ages for the separation of ophiuroid families obtained by O'Hara, et al.^5^ from 285 kb of sequence from 1552 exons and 11 fossil-based reliable date calibrations. By COI, the Ophiocomoidea (represented by *Ophiocomella*) and the Ophiodermatoidea (represented by *Ophiarachna, Ophiarachnella* and *Ophioderma*) were estimated by the COI chronogram to have branched from each other between the Paleocene and the Oligocene (median: 49.2 MYA, 95% HPD: 86.1–25.5 MYA). The Ophiomyxidae (*Ophiarachna*) were estimated as having split from the Ophiodermatidae (*Ophiarachnella, Ophioderma*) at 37.6 MYA (95% HPD: 64.8–18.9 MYA). According to the more reliable analysis by O’Hara et al. these separations between superfamilies and families occurred much earlier, between the Triassic and the Cretaceous 250–100 MYA.

More recent dates estimated on the basis of COI, which may be more accurate as they involve fewer multiple hits, suggest that *Ophioderma* clades that lead to extant species are older than those of extant congeneric species in other echinoderm classes. In *Ophioderma,* lineages terminating in extant species coalesce in the Oligocene; much of the cladogenic activity has occurred 27–10 MYA between the middle-Oligocene and early Miocene (Fig. 3). In most phylogenies of extant echinoids, COI lineages of extant species within genera coalesce more recently: ~ 0.5 MYA in *Paracentrotus*^81^*,* ~ 3 MYA in *Tripneustes*^82^ and also in *Lytechinus*^83^, ~ 4 MYA in *Echinometra*^84^, *Heliocidaris*^85^ and in *Arbacia*^86^, ~ 5 MYA in *Eucidaris*^87^, ~ 5.5 MYA in *Mellita*^88^ (but ~ 14 MYA in *Diadema*^89^, and ~ 15 MYA in *Encope*^90^). The limited number of dated phylogenies of asteroid genera also suggest that lineages within a genus coalesce more recently than they do in *Ophioderma.* In *Asterias* they only go back to ~ 3.5 MYA^91^ and in *Leptasterias* ~ 8 MYA^92^. In holothurians the most distant species of *Stichopus* go back to 4.6–8.8 MYA^93^, but those of the immense, paraphyletic genus *Holothuria* are quite old, coalescing in the Triassic, some 240 MYA^94^. The case of *Holothuria* illustrates that taxonomic decisions as to the size of a genus will influence the appearance of antiquity of the species it contains. However, it is not likely that the species of *Ophioderma* appear to be old only because the genus has been too broadly defined, because there are no obvious morphological or molecular discontinuities that would suggest that it should be subdivided.

Whether this longevity of lineages is characteristic of ophiuroids in general or of *Ophioderma* in particular remains to be determined. The phylogeny of Ophiocomidae by O'Hara, et al.^28^ reveals that their genera are also old. In this family, the genus that evolved most recently dates back to the Paleogene, 30 MYA. The persistence of ophiuroid lineages terminating to the present time suggests that this class of echinoderms may suffer a lower rate of extinction than echinoids. This may be part of the reason that they contain more than double the number of extant species than echinoids.

Contrary to conclusions from previous studies that the ophiuroid mitochondrial mitogenome in general and the COI gene in particular evolve faster than that of other classes of echinoderms^95,96^, COI substitution rate in *Ophioderma,* as calibrated from the age of the completion of the Isthmus of Panama, is three times slower than the average rate of similarly dated echinoids. The separation between the geminate species *O. teres* and *O. holmesii* dated from BEAST, occurred 3.5 MYA, and the genetic distance between the two species was 4.02%. The separation of *O. elaps* from *O.* aff. *pentacanthum* was also dated at 3.5 MYA and the genetic distance was 4.95%. Thus, *Ophioderma* divergence rate in COI has proceeded at approximately 1.15–1.41% per MY. Roy and Sponer^97^ estimated the rate of COI divergence in *Ophiactis* to be 0.87% per MY. In echinoids, the average rate of COI divergence in six genera summarized by Lessios^78^ is 3.66% (range 2.90–4.50%) per MY. Since that publication, a higher rate of 7.85% per MY has been found in *Mellita*^88^ and a much slower rate of 0.23% in *Encope*^90^, illustrating that even within a single family rates of substitution can vary by an order of magnitude, but preserving the echinoid mean at 3.76% per MY. With the exception of Roy and Sponer ^97^ and of Richards, et al.^25^, previous studies of evolution in ophiuroids based on COI^12,14,17,18,98^ relied on rate calibrations from echinoids, as the echinoderm group in which the calibrations were most extensively determined at the time that the studies were conducted. The dates in these studies are in need of revision, as are some of the conclusions based on them. Given the substitution rate of COI in *Ophioderma*, the clades (now different species) of *O. longicaudum* did not begin separating at the time of Pleistocene glaciations after 2.4 MYA as Boissin, et al.^14^ estimated, but more likely at about 7 MYA, before the Messinian crisis^99^. Assuming that class-specific calibrations provide better estimates than phylum-based estimates, and applying the *Ophioderma* calibration to studies of other ophiuroid genera, the sister clades of *Ophiarachnella, Ophiopeza* and *Ophiolepis* discovered by Hoareau, et al.^18^ in the southern Indian Ocean did not separate between 1.6 and 3.9 MYA, but rather between 4.2 and 11.7 MYA. The divergence between intertidal and subtidal populations of *Acrocnida brachiata* occurred closer to 10 MYA instead of 3.5^98^ or 5 MYA^12^, adding evidence in favor of recognizing the intertidal form as a separate species^100^. Similarly, divergence between lineages of European *Ophiothrix* occurred 14–22 MYA, rather than the Miocene–Pliocene transition 4.8–7.5 MYA^10^.

## Conclusions

The COI phylogeny of the species of *Ophioderma* is far from the last word on the reconstruction of the relationships between its species, but it does illustrate that several undescribed species may be present, and that, dated with calibrations specific to this genus, lineages coalesce farther back in time than those of the studied genera of echinoids, asteroids and holothuroids.

## Materials and methods

### Collection of specimens

**Figure 4:**
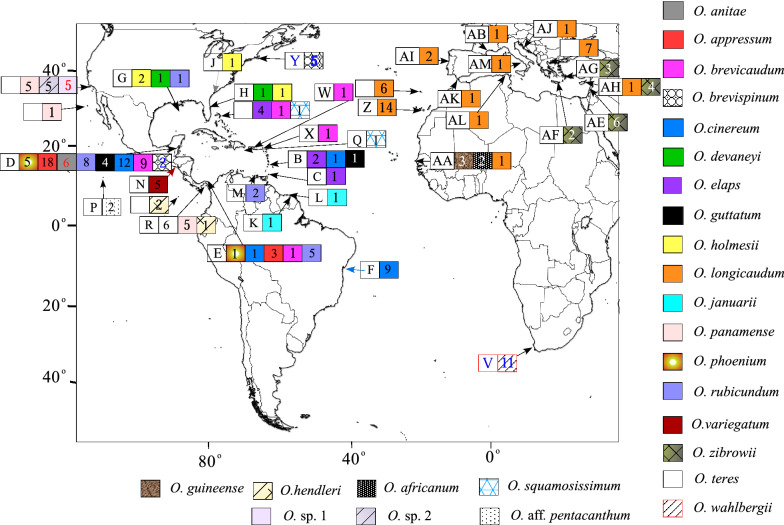
Collection localities of specimens of *Ophioderma* used in this study. Colors indicate the species as determined from their morphology; letters indicate localities, numbers indicate sample size of each species. ***O. anitae:*** D: Belize (Carrie Bow Cay). ***O. appressum:*** E: Panama (Bocas del Toro), D: Belize (Carrie Bow Cay). ***O. brevicaudum:*** E: Panama (Bocas del Toro), D: Belize (Carrie Bow Key), W: Puerto Rico (off Isla Maguayez), Σ: Bahamas (San Salvador), X: US Virgin Islands (St. Croix). ***O. brevispinum:*** D: Belize (Norval Cay), Y: Massachusetts (Waquoit Bay, Cape Cod). ***O. cinereum***: D: Belize (Carrie Bow Cay, Twin Cays), B: Saint Vincent, E: Panama (Portobelo), F: Salvador, Brazil. ***O. devaneyi:*** G: Gulf of Mexico (S. of Galveston), H: Florida (NE of Vero Beach). ***O. elaps:*** Σ: Bahamas (San Salvador, Eleuthera, Rum Cay), B: Saint Vincent, C: Barbados. ***O. guttatum***: D: Belize (Carrie Bow Cay), B: Saint Vincent. ***O. hendleri*** Θ: Isla del Coco, R: Bay of Panama (Islas Perlas). ***O. holmesii***: H: Florida (NE off Vero Beach), G: Gulf of Mexico. J: South Carolina (Edisto Island). ***O. januarii***: K: Guyana (Maroni), L: Suriname (Paramaribo). ***O. sp. 1:*** Γ: Southern California (Anacapa Island, San Clemente Island, Santa Barbara Island, Santa Catalina Island), ***O. sp. 2:*** Γ: South California (Corona del Mar, San Clemente Island, Santa Catalina Island), Δ: Isla Guadalupe, Mexico. ***O. panamense:*** R: Bay of Panama (Perlas Islands). ***O. phoenium***: D: Belize (Carrie Bow Cay), E: Panama (Portobelo). ***O. rubicundum:*** E: Panama (Bocas del Toro, Portobelo), G: Gulf of Mexico, M: Curacao (Habitat Reef, W. Coast), D: Belize (Carrie Bow Cay). ***O. variegatum***: N: Off El Salvador. ***O. aff. pentacanthum:*** P: Clipperton Island. ***O. squamosissimum***: Q: Navassa Island, Σ: Bahamas (San Salvador). ***O. teres***: R: Bay of Panama (Isla Taboguilla, Islas Perlas). ***O. wahlbergii***: V: South Africa (False Bay, Cape of Good Hope). ***O. longicaudum***: Z: Gran Canaria, Ψ: Madeira, Ω: North Aegean (Kassandra Peninsula). Two-letter codes starting with “A” in *O. longicaudum, O. zibrowii, O. africanum* and *O. guineense* are localities of sequences obtained by Boissin, et al.^14^ with species names designated by Stöhr et al.^34^^.^ AA: Senegal (Dakar), AB: France (Marseille), AE: Lebanon, AF: Crete, AG: Dodekanese (Symi, Rhodes), AH: Cyprus, AI: Portugal (Algarve), AJ: Croatia, AK: African Spain (Ceuta), AL: Tunisia, AM: Italy (Naples). Detailed location data are listed in Supplementary Table S2. See text for explanation of taxa designated as *O.* sp,1, *O.* sp,2, and *O.* aff. *pentacanthum*. Map outline downloaded from http://woodshole.er.usgs.gov/mapit/.

We sampled a total of 185 individuals of 21 species of *Ophioderma* from 25 localities (Fig. 4) either collected by us, donated at our request, or available in the Natural History Museum of Los Angeles County. To the set of our data, we added 16 Cytochrome Oxidase I (COI) sequences of *O. longicaudum* from Boissin, et al.^14^, 3 to 5 from each of the six clades of COI they identified. Their clade L1 (C3 in Weber et al^101^) corresponds to *O. longicaudum*^34^, their clades L2, L3 and L4 (C2, C5 and C6 in Weber et al.^101^) correspond to *O. zibrowii*, clade L5 (C2) is present in *O. africanum,* and L6 (C1) in *O. guineense,* although the species name of Mediterranean specimens that share this clade is unclear (see introduction). Sequences of *O. longicaudum* from the Canary Islands and from Madeira (GenBank Accession numbers FJ716117, FJ716121, FJ716122, JN603483-JN603485, JN603517- JN603525, JN603556) that appeared in Boissin, et al.^14^ had been obtained by us for the present study. Of the species of *Ophioderma* that are currently regarded as valid in the World Register of Marine Species^1^, we were unable to either obtain specimens or to amplify DNA from *O. besnardi, O. ensiferum*, *O. divae* and *O. pallidum* from the western Atlantic and from *O. pentacanthum, O. vansyoci, O. peruanum,* and *O. sodipallaresi* from the eastern Pacific. As outgroups we included one specimen of *Ophiarachnella petersi* from the Bahamas, one of *Ophiarachna incrassata* from the Philippines, and one of *Ophiocomella* (previously *Ophiocoma*) *pumila* from the Atlantic coast of Panama. Samples were preserved in 95% ethanol or in high-salt DMSO buffer^102^.

### DNA extraction and sequencing

Genomic DNA was extracted from pieces of the arms of the ophiuroids by proteinase K digestion^103^, or using Qiagen DNeasy Blood and Tissue®, or Gentra Puregene Tissue® Kits, or Lucigen “Quick Extract” protocol®, or Promega Wizard Plus Purification System®. Amplifications of a 625 bp fragment of the COI region of mtDNA were carried out with primers CO1-f 5 CCTGCAGGAGGAGGAGAYCC or OphCOI-For 5' CAACAYYTATTYTGRTTYTTYGG in the forward direction, and CO1-a 5' AGTATAAGCGTCTGGGTAGTC or OphCOI-Rev 5' CCTARRAARTGTTGWGGGAARAA or CO1-TR1 5' GGCATTCCAGCTAGTCCTARAA in the reverse direction. PCR amplification was performed in 50 μL of PCR reaction mixture A (0.3 units of Promega Flexi Go Taq® , 2.5 μL of 5X colorless buffer, 0.625 μ1 of 10 μM of each primer, 1.25 μL of 8 mM dNTPs, 1.25 μL of 25 mM MgC1_2_) or reaction mixture B (0.4 units of Invitrogen Platinum Taq® , 1.25 μL of 10X buffer, 0.625 μL of 10 μM of each primer, 2 μL of 0.8 mM dNTPs, 0.625 μL of 100% Dimethyl Sulfoxide, 0.75 μL of 50 mM MgC1_2_). The samples were heated to 96 °C for 5 s, then cycled 39 times through 94° C for 30 s, 50° C for 45 s, 72° C for 60 s, followed by 5 m in 72 °C and 5 m in 10 °C. The PCR products were cleaned with the ExoSap-IT® kit (USBCorporation), then cycle sequenced in both directions using the amplification primers and electrophoresed in an ABI3130 or and ABI3500 automated sequencer. Attempts to amplify nuclear markers, the i51 intron^104^ and an Actin-2 intron, produced unreliable amplifications and inconsistent results in different extractions; these data were not used.

### Phylogenetic analyses

We eliminated redundant haplotypes and the outgroups from the set of data and then used Posada’s^105^ jModelTest v. 0.1.1 program to determine the simplest model of mitochondrial DNA that produced the best fit of the data to the tree, based on the AIC criterion^106^. This was the General Time-Reversible model^107^ with a gamma correction (α = 0.907) and a proportion of invariable sites (P = 0.5480). After adding the outgroups, we used this model and two algorithms to reconstruct the mitochondrial phylogeny of the species of *Ophioderma*. We performed phylogenetic reconstruction by Bayesian Inference (BI) in MrBayes v.3.2.2^108^ and by Maximum Likelihood (ML) in RAxML v. 8.2.6^109^ (without the invariable site correction). The ML analysis was run in the CIPRES Gateway^110^. We used the options for rapid bootstraps and automatic halting. Support values for the nodes were estimated from 504 bootstraps. In MrBayes we employed the models suggested by jModelTest, but let the program estimate the parameters. Mr. Bayes was run in 2 chains for 3 × 10^7^ steps, which allowed the average standard deviation of split frequencies to fall below 0.01, and the potential scale reduction factor to be equal to 1.00. Convergence was also determined in multiple runs, which produced the same topology. Nodes that received < 80% support in ML and < 0.9 in BI were collapsed.

To estimate dates of divergence between major clades we used BEAST v. 1.10.4^111^. The program was given the fully resolved tree produced by RAxML, which was compatible with the MrBayes tree. Operators causing topology searches (“SubtreeSlide”, “Narrow Exchange”, “Wide exchange”, “WilsonBalding”) were turned off to force BEAST to place time estimates on the nodes of the ML tree, but BEAST was allowed to estimate branch lengths. To calibrate rate of divergence, the separation between Atlantic and Pacific haplotypes of two pairs of amphi-American sister species with the least divergence between their members, *O. holmesii-O. teres* and *O. elaps-O*. aff. *pentacanthum* (see results), was given an offset of 3 million years (MY) in a Lognormal Uncorrelated Relaxed clock. Three MY is the generally accepted approximate date of the completion of the Central American Isthmus^78,79,112,113^. However, as there are claims that there were intermittent closures starting at approximately 13 MYA^113,114^, the priors for these calibration points were set with exponential distributions, ranging from 3 to 13 MY. Three separate runs of 10^7^ steps each, recording every 1000th tree were performed. Logs from the three runs were combined in LogCombiner v. 1.10.4 after removing the first 10^3^ trees from each run and viewed in Tracer v. 1.6 to verify that there were no trends and that effective sample size (ESS) values for all estimated parameters was > 231.

Maximum likelihood composite genetic distances^115^, taking into account differences in composition bias^116^, were calculated in MEGA v. 7.0.20^117^ with gamma corrections as estimated by jModelTest.

## Supplementary Information

Supplementary Information 1.

## Data Availability

The sequence data generated during the current study are available in GenBank (https://www.ncbi.nlm.nih.gov/genbank/) under accession numbers shown in Supplemental Table S2.
